# Molecular and Biochemical Basis of Fluoroquinolones-Induced Phototoxicity—The Study of Antioxidant System in Human Melanocytes Exposed to UV-A Radiation

**DOI:** 10.3390/ijms21249714

**Published:** 2020-12-19

**Authors:** Justyna Kowalska, Klaudia Banach, Jakub Rok, Artur Beberok, Zuzanna Rzepka, Dorota Wrześniok

**Affiliations:** Department of Pharmaceutical Chemistry, Faculty of Pharmaceutical Sciences in Sosnowiec, Medical University of Silesia in Katowice, Jagiellońska 4, 41-200 Sosnowiec, Poland; jkowalska@sum.edu.pl (J.K.); kbanach@sum.edu.pl (K.B.); jrok@sum.edu.pl (J.R.); abeberok@sum.edu.pl (A.B.); zrzepka@sum.edu.pl (Z.R.)

**Keywords:** lomefloxacin, moxifloxacin, phototoxicity, superoxide dismutase, catalase, glutathione peroxidase

## Abstract

Phototoxicity of fluoroquinolones is connected with oxidative stress induction. Lomefloxacin (8-halogenated derivative) is considered the most phototoxic fluoroquinolone and moxifloxacin (8-methoxy derivative) the least. Melanin pigment may protect cells from oxidative damage. On the other hand, fluoroquinolone–melanin binding may lead to accumulation of drugs and increase their toxicity to skin. The study aimed to examine the antioxidant defense system status in normal melanocytes treated with lomefloxacin and moxifloxacin and exposed to UV-A radiation. The obtained results demonstrated that UV-A radiation enhanced only the lomefloxacin-induced cytotoxic effect in tested cells. It was found that fluoroquinolones alone and with UV-A radiation decreased superoxide dismutase (SOD) activity and *SOD1* expression. UV-A radiation enhanced the impact of moxifloxacin on hydrogen peroxide-scavenging enzymes. In turn, lomefloxacin alone increased the activity and the expression of catalase (CAT) and glutathione peroxidase (GPx), whereas UV-A radiation significantly modified the effects of drugs on these enzymes. Taken together, both analyzed fluoroquinolones induced oxidative stress in melanocytes, however, the molecular and biochemical studies indicated the miscellaneous mechanisms for the tested drugs. The variability in phototoxic potential between lomefloxacin and moxifloxacin may result from different effects on the antioxidant enzymes.

## 1. Introduction

Adverse cutaneous drug reactions frequently occur in clinical practice. Skin lesions account for approximately 45% of all adverse drug effects [1]. It has been reported that a lot of widely used drugs cause photosensitivity reactions [2].

Phototoxicity, a type of photosensitivity, is indicated when a photosensitizing drug or a drug metabolite absorbs photons and becomes an excited molecule [3,4]. A photosensitizer molecule in the singlet excited state reacts directly with cellular macromolecules and organelles, leading to cell damage. Moreover, a molecule may also be in the triplet excited state which is more stable and has a much longer lifetime [5,6]. The phototoxic action of a molecule in the triplet excited state may result from the following events: (i) formation of singlet oxygen through the energy transfer from the excited photosensitizer to molecular oxygen, or (ii) production of reactive oxygen species (ROS) superoxide anion, hydrogen peroxide, or hydroxyl radical through electron or hydrogen transfer [7]. ROS participate in the oxidative damage of important cellular biomolecules, i.e., nucleic acids, proteins, or lipids [3,5].

Fluoroquinolones (FQs) are a class of antibacterial agents with potent bactericidal activity against a broad spectrum of clinically important pathogens. Their usage can be limited by, among others, cutaneous adverse reactions. Treatment with FQs is often related to the risk of photoallergy, phototoxicity, and photocarcinogenesis. Chemically, fluoroquinolones are fluorinated derivatives of nalidixic acid [8,9,10]. It has been demonstrated that a substituent at the C-8 position of a 4-quinolone group responds to phototoxic properties. Analogs with a halogen atom at the C-8 position are unstable under ultraviolet radiation and they have higher phototoxic potency than 8-methoxy derivatives [11,12,13,14,15].

The fluoroquinolones with a halogen atom at the C-8 position undergo photodegradation, including dehalogenation and the formation of aryl cations [16,17]. Aryl cations are very reactive in the excited triplet state. They directly interact with other cellular components [5]. Moreover, aryl cations react with water and molecular oxygen, resulting in the production of hydrogen peroxide or hydroxyl radicals in the Fenton reaction [18].

The melanin pigment, produced and stored in melanocytes, may neutralize free radicals and thus provides protection to cells against oxidative stress. Moreover, melanin interacts with xenobiotics and modifies their toxicity to pigmented tissues [19]. Previously, we demonstrated that FQs [20,21,22,23] and other drugs with photosensitivity potential, such as ketoprofen [24], tetracyclines [25], and phenothiazines [26,27], bound with melanin and affected homeostasis of melanocytes.

The study aimed to examine the effect of lomefloxacin and moxifloxacin (8-halogenated and 8-methoxy fluoroquinolone, respectively) on the antioxidant defense system in normal human epidermal melanocytes exposed to UV-A radiation.

## 2. Results

### 2.1. The Impact of Fluoroquinolones and UV-A Radiation on Cell Viability

**Figure 1:**
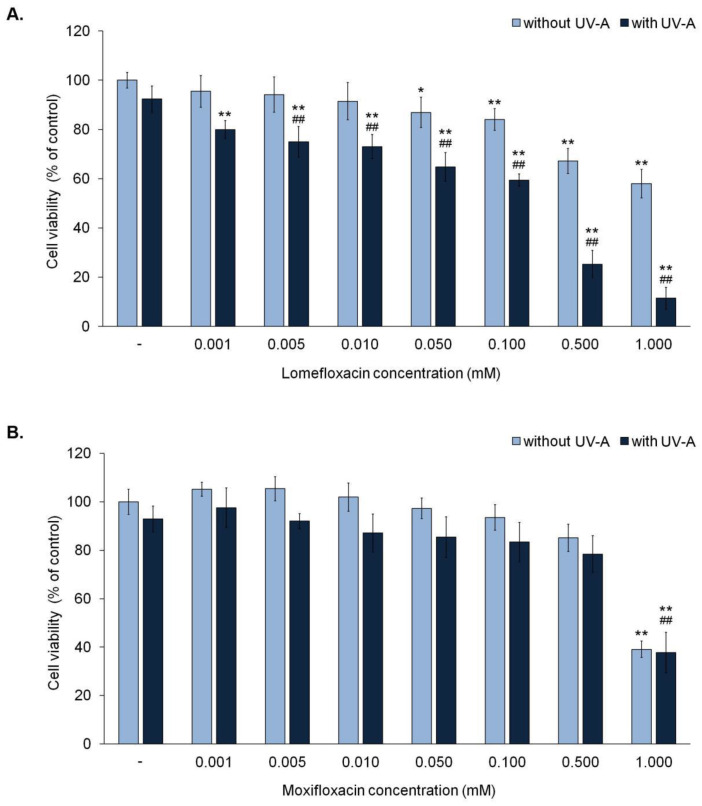
Fluoroquinolones affected the viability of melanocytes exposed to UV-A radiation. The cells were treated with increasing concentrations (0.001–1.0 mM) of lomefloxacin (**A**) and moxifloxacin (**B**). Data were presented as % of the control. * *p* < 0.05, ** *p* < 0.01 vs. control; ## *p* < 0.01 vs. non-treated cells exposed to UV-A radiation.

The influence of fluoroquinolones on melanocyte viability was evaluated by the WST-1 test. Lomefloxacin did not affect cell viability in a concentration range from 0.001 mM to 0.01 mM, whereas in concentrations of 0.05 mM, 0.1 mM, 0.5 mM, and 1.0 mM it caused a decrease in cell viability by about 13%, 16%, 33%, and 42%, respectively (Figure 1A). Moxifloxacin showed a significant cytotoxic effect only in the highest analyzed concentration. The incubation of melanocytes with moxifloxacin in a concentration of 1.0 mM caused a reduction in cell viability of about 61% (Figure 1B).

The UV-A radiation alone did not influence cell viability, whereas it caused an increase in the cytotoxicity effect of lomefloxacin. Simultaneous exposure of melanocytes to UV-A radiation and lomefloxacin in concentrations 0.001 mM, 0.005 mM, 0.01 mM, 0.05 mM, 0.1 mM, 0.5 mM, and 1.0 mM decreased cell viability by approx. 20%, 21%, 25%, 27%, 35%, 40%, 77%, and 89%, respectively. UV-A irradiation did not increase the cytotoxic effect of moxifloxacin. A significant decrease in cell viability, i.e., 62%, was observed only after simultaneous exposure of cells to UV-A radiation and moxifloxacin in a concentration of 1.0 mM.

Taking into consideration the observed cytotoxic and phototoxic effects of fluoroquinolones, the estimation of the antioxidant defense system status in human normal melanocytes was determined by the use of 0.005 mM, 0.05 mM, and 0.5 mM lomefloxacin concentrations and 0.01 mM, 0.1 mM, and 1.0 mM moxifloxacin concentrations. Moreover, the impact of FQs and UV-A radiation on the expression of antioxidant enzymes was examined using lomefloxacin in a concentration of 0.05 mM and moxifloxacin in a concentration of 0.1 mM.

### 2.2. The Effect of Fluoroquinolones and UV-A Radiation on Activity and Expression of Superoxide Dismutase

Superoxide dismutase (SOD) plays an essential role in the first line of antioxidant defense. It catalyzes the conversion of the highly reactive superoxide anion to the less reactive hydrogen peroxide. In human cells, there are two isoforms of SOD: cytosolic SOD1 and mitochondrial SOD2 [28].

**Figure 2:**
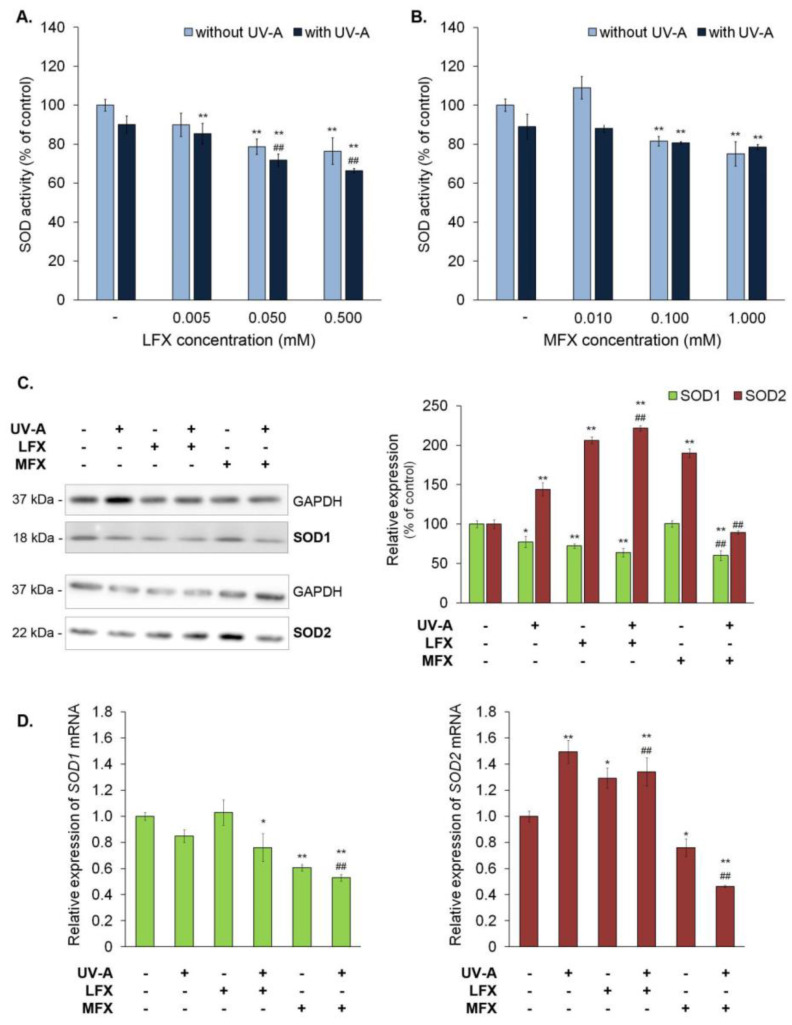
Fluoroquinolones decreased superoxide dismutase activity in melanocytes through down-regulation expression of superoxide dismutase isoform 1. UV-A radiation (720 μW/cm^2^) potentiated the influence of lomefloxacin only on the activity of superoxide dismutase. The activity of superoxide dismutase was determined by the use of lomefloxacin (LFX) in concentrations of 0.005 mM, 0.05 mM, and 0.5 mM (**A**) and moxifloxacin (MFX) in concentrations of 0.01 mM, 0.1 mM, and 1.0 mM (**B**). The protein (**C**) and mRNA (**D**) levels of superoxide dismutase isoforms 1 and 2 were conducted for lomefloxacin in a concentration of 0.05 mM and moxifloxacin in a concentration of 0.1 mM. The bar graph represents mean ± SD from three independent experiments. * *p* < 0.05, ** *p* < 0.01 vs. control; ## *p* < 0.01 vs. non-treated cells exposed to UV-A radiation.

The analysis of superoxide dismutase activity revealed that lomefloxacin in concentrations of 0.05 mM and 0.5 mM caused a decrease in SOD activity by approximately 21% and 24%, respectively (Figure 2A). Similarly, moxifloxacin in concentrations of 0.1 mM and 1.0 mM decreased SOD activity by about 18% and 25%, respectively (Figure 2B).

UV-A irradiation of cells treated with moxifloxacin in concentrations of 0.1 mM and 1.0 mM resulted in a reduction in SOD activity of about 20% when compared with the control. The obtained results indicated that UV-A radiation potentiated the impact of lomefloxacin on SOD activity. The simultaneous exposure to UV-A radiation and lomefloxacin in concentrations ranging from 0.005 mM to 0.5 mM resulted in a decrease in enzyme activity by about 15–34%. Moreover, there was a significant reduction in SOD activity in melanocytes exposed to UV-A radiation and lomefloxacin (in concentrations of 0.05 mM and 0.5 mM) when compared to the non-treated cells exposed to UV-A radiation.

It was also observed that the activity of SOD and the expression of *SOD1* were changed in the same manner. The Western blot analysis (Figure 2C) showed a decrease in the SOD1 level by about 23% in melanocytes exposed to UV-A radiation alone. In turn, lomefloxacin alone reduced SOD1 by about 28%. The simultaneous exposure of melanocytes to UV-A radiation and lomefloxacin resulted in a significant reduction in the SOD1 level, i.e., of approximately 36%. Moreover, melanocytes exposed to UV-A radiation and lomefloxacin had a significantly lower level of *SOD1* mRNA (by approximately 0.25) when compared with the control (Figure 2D). In melanocytes treated with moxifloxacin alone, a reduction in the *SOD1* mRNA level of 0.39 was observed. The obtained results revealed a significant decrease in the protein (by 40%) and mRNA (by 0.36) level of SOD1 in cells exposed to UV-A radiation and moxifloxacin when compared with non-treated cells.

The evaluation of *SOD2* expression demonstrated that UV-A irradiation caused an increase in protein and mRNA levels. The RT-qPCR and Western blot analysis also indicated the increased SOD2 expression in melanocytes exposed to lomefloxacin alone and simultaneously with UV-A radiation. In turn, the exposure of cells to UV-A radiation and moxifloxacin resulted in a decrease in the mRNA level of *SOD2*.

### 2.3. The Effect of Fluoroquinolones and UV-A Radiation on Activity and Expression of Catalase

Catalase (CAT) is the major antioxidant enzyme that protects melanocytes from oxidative damage by hydrogen peroxide. This enzyme catalyzes the dismutation of hydrogen peroxide to water and molecular oxygen [28,29].

**Figure 3:**
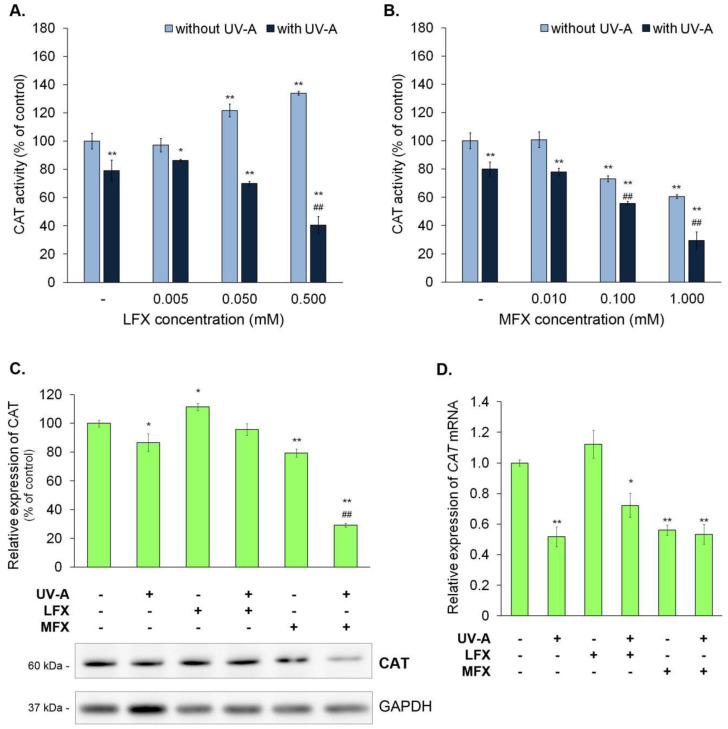
Lomefloxacin and moxifloxacin had different effects on catalase activity and expression in melanocytes. UV-A radiation (720 μW/cm^2^) increased the impact of moxifloxacin and reversed that of lomefloxacin on catalase. The activity of catalase was determined by the use of lomefloxacin (LFX) in concentrations of 0.005 mM, 0.05 mM, and 0.5 mM (**A**) and moxifloxacin (MFX) in concentrations of 0.01 mM, 0.1 mM, and 1.0 mM (**B**). The protein (**C**) and mRNA (**D**) levels of catalase were conducted for lomefloxacin in a concentration of 0.05 mM and moxifloxacin in a concentration of 0.1 mM. The bar graph represents mean ± SD from three independent experiments. * *p* < 0.05, ** *p* < 0.01 vs. control; ## *p* < 0.01 vs. non-treated cells exposed to UV-A radiation.

The obtained results showed that lomefloxacin in a concentration of 0.005 mM did not influence CAT activity (Figure 3A), whereas in concentrations of 0.05 mM and 0.5 mM it caused an increase in enzyme activity to approximately 122% and 134%, respectively. Analogously, moxifloxacin in the lowest analyzed concentration of 0.01 mM had no effect on catalase activity. However, in contrast with lomefloxacin, moxifloxacin in concentrations of 0.1 mM and 1.0 mM reduced the enzyme activity by about 27% and 39%, respectively (Figure 3B).

A significant decrease in CAT activity was observed in all irradiated samples. UV-A irradiation of non-treated melanocytes resulted in a reduction in CAT activity of about 20% and the simultaneous exposure to UV-A radiation and lomefloxacin in concentrations of 0.005 mM, 0.05 mM, and 0.5 mM caused the inhibition of catalase activity by approximately 14%, 30%, and 59%, respectively, when compared with the control. Moreover, UV-A irradiation of melanocytes treated with lomefloxacin in a concentration of 0.5 mM significantly decreased CAT activity (by 38%), compared with the non-treated cells exposed to UV-A radiation. In melanocytes irradiated by UV-A radiation and treated with moxifloxacin in concentrations of 0.01 mM, 0.1 mM, and 1.0 mM, activity of CAT was decreased by approximately 22%, 44%, and 71%, respectively, when compared with the control. Otherwise, the simultaneous exposure to UV-A radiation and moxifloxacin (in concentrations of 0.1 mM and 1.0 mM) resulted in a significant decrease in enzyme activity when compared with non-treated cells exposed to UV-A radiation.

The alterations of CAT activity corresponded to the changes in CAT expression examined by Western blot (Figure 3C) and RT-qPCR analysis (Figure 3D). Lomefloxacin alone caused a significant increase of about 12% in the protein level of catalase, whereas UV-A radiation alone and moxifloxacin alone decreased the protein level of CAT by about 13% and 21%, respectively, and the mRNA level by 0.48 and 0.44, respectively. The melanocytes exposed to UV-A radiation and lomefloxacin were shown to have a significantly decreased mRNA level of catalase by 0.28, compared with the control. The irradiation of cells treated with moxifloxacin resulted in a reduction in CAT protein (by 71%) and mRNA level (by 0.47), compared with the control. Moreover, these cells had a decreased CAT protein level compared with non-treated cells exposed to UV-A radiation.

### 2.4. The Effect of Fluoroquinolones and UV-A Radiation on Activity and Expression of Glutathione Peroxidase

Glutathione peroxidase (GPx) is a selenium-containing enzyme that converts hydrogen peroxide to water using glutathione. In humans, there are eight isoforms of GPx, and GPx isoform 1 is found in most tissues [28,30].

**Figure 4:**
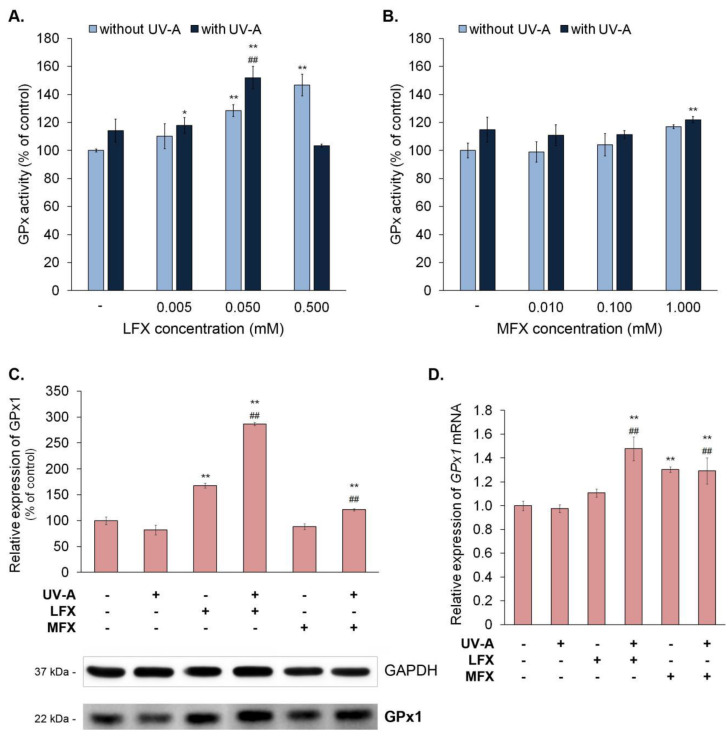
Lomefloxacin increased the activity and expression of glutathione peroxidase in melanocytes, whereas moxifloxacin had no effect on the enzyme. UV-A radiation (720 μW/cm^2^) modified the impact of fluoroquinolones on glutathione peroxidase. The activity of glutathione peroxidase was determined by the use of lomefloxacin (LFX) in concentrations of 0.005 mM, 0.05 mM, and 0.5 mM (**A**) and moxifloxacin (MFX) in concentrations of 0.01 mM, 0.1 mM, and 1.0 mM (**B**). The protein (**C**) and mRNA (**D**) levels of glutathione peroxidase isoform 1 were conducted for lomefloxacin in a concentration of 0.05 mM and moxifloxacin in a concentration of 0.1 mM. The bar graph represents mean ± SD from three independent experiments. * *p* < 0.05, ** *p* < 0.01 vs. control; ## *p* < 0.01 vs. non-treated cells exposed to UV-A radiation.

The GPx activity analysis showed that moxifloxacin in concentrations of 0.01 mM, 0.1mM, and 1.0 mM did not significantly influence the activity of the enzyme (Figure 4B). In turn, lomefloxacin in concentrations of 0.05 mM and 0.5 mM increased the enzyme activity by about 28% and 47%, respectively (Figure 4A).

The simultaneous exposure of melanocytes to UV-A radiation and lomefloxacin in concentrations of 0.005 mM and 0.05 mM resulted in an increase in GPx activity of about 18% and 52% when compared with the control. Moreover, the irradiation of cells treated with lomefloxacin in a concentration of 0.05 mM caused a rise in enzyme activity when compared with non-treated cells exposed to UV-A radiation. In the case of moxifloxacin, the significant change in GPx activity was observed only in melanocytes exposed to the drug in a concentration of 1.0 mM and UV-A radiation. The activity increased by about 22% when compared with the control.

The observed changes in GPx1 expression examined by Western blot and RT-qPCR techniques were demonstrated in Figure 4C,D. Lomefloxacin alone caused an increase in the protein level of GPx1 of 68%. The simultaneous exposure of cells to UV-A radiation and lomefloxacin resulted in a significant increase in the protein (by 186%) and mRNA level (by 0.48) when compared with the control. Moreover, similar changes in GPx1 expression were observed compared with UV-exposed cells. The obtained results indicated that moxifloxacin caused an increase in the mRNA level of 0.3. The melanocytes exposed to UV-A radiation and moxifloxacin were shown to have increased the protein level of GPx1 (by 21%) and the mRNA level (by 0.29) when compared with the control. These cells had also increased the GPx1 protein and mRNA level when compared with non-treated melanocytes exposed to UV-A radiation.

## 3. Discussion

FQs induce skin and ocular phototoxicity [15,18]. The presence of a melanin biopolymer is a well-known feature of the skin and eyes. There are two basic types of melanin in the skin: brown-black eumelanin and yellow-red pheomelanin. The color of the skin is determined by the ratio of eumelanin to pheomelanin. Eumelanin has high photoabsorbing capacity and neutralizes ROS, whereas pheomelanin is photolabile under sunlight and is involved in superoxide generation [31,32,33]. Additionally, melanin binds organic biomolecules and xenobiotics, such as drugs. Melanin–drug complex formation may lead to the accumulation of drugs in pigmented cells and can modify their toxicity to melanin-containing tissues [34,35]. Previously, we indicated that FQs bound to melanin pigment. It was noticed that FQ derivatives differed in their capacity to bind to this biopolymer. An analysis of drug binding to melanin by the use of the Scatchard plot has shown that more than one class of binding sites is implicated in the drug–melanin complex formation: for lomefloxacin strong binding sites (n_1_) with the association constant K_1_ ~6 × 10^5^ M^−1^ and weak binding sites (n_2_) with the association constant K_2_ ~7 × 10^2^ M^−1^, and for moxifloxacin strong binding sites (n_1_) with the association constant K_1_ ~1.4 × 10^4^ M^−1^ and weak binding sites (n_2_) with the association constant K_2_ ~6.9 × 10^2^ M^−1^. The total number of binding sites (n_1_ + n_2_) was 0.92 µmol lomefloxacin and 0.39 µmol moxifloxacin per 1 mg melanin, indicating that the least and the most phototoxic FQ had miscellaneous abilities to form complexes with melanin, which may explain differences in the phototoxic potential of the tested FQ derivatives [22,23].

Drug-induced phototoxicity is associated with oxidative stress [2]. The overproduction of ROS causes damage to cellular components, such as nucleic acids, lipids, and proteins. The most important ROS generated in cells include singlet oxygen (^1^O_2_), superoxide anion radicals (O_2_^•−^), hydrogen peroxide (H_2_O_2_), and hydroxyl radicals (^•^OH) [36]. Oxidative damage to DNA results from modifications of bases and deoxyribose to strand breakage. It also leads to changes in the binding of transcription factors and causes gene mutations. Fragmentation, dimerization, covalent cross-linking, and abnormal aggregation of proteins and enzyme inactivation occur as a consequence of the attack of ROS on structural and enzymatic proteins. Moreover, ROS overproduction leads to the peroxidation of lipids. Oxidative modifications of lipids result in increased fluidity and permeability of organellar and cellular membranes, as well as the generation of lipid-derived radicals that interact with cellular biomolecules, causing cell injury [37,38].

Our results showed that UV-A radiation did not increase the cytotoxic effect of moxifloxacin to normal melanocytes. The simultaneous exposure of cells to UV-A radiation and moxifloxacin caused similar changes in cell viability as exposure to moxifloxacin alone. These observations indicated that moxifloxacin had no significant phototoxic effect on melanocytes. In the case of lomefloxacin, we observed an increase in the cytotoxicity of the drug to melanocytes under UV-A irradiation. The exposure to UV-A radiation caused significant loss in the viability of cells treated with lomefloxacin in all tested concentrations. These results were consistent with the data of other studies. It has been demonstrated that FQ analogs with a methoxy group at the C-8 position, such as moxifloxacin, are stable under UV-A radiation, and thus have low phototoxic potential [13,14]. In contrast, 8-halogenated FQs, such as lomefloxacin, rapidly undergo photodegradation upon UV-A irradiation, resulting in reactive aryl cation generation and cellular damage. Therefore, FQs with a halogen atom at the C-8 position have high phototoxic activity [16,17].

It was reported that FQs irradiated with UV-A induced the generation of ROS such as superoxide radicals and hydrogen peroxide [39,40]. Therefore, we examined the influence of lomefloxacin and moxifloxacin on the activity and the expression of ROS-neutralizing enzymes in melanocytes exposed to UV-A radiation.

Since oxidative stress is implicated in molecular and cellular damage, cells posses a complex antioxidant system. Defense mechanisms against redox imbalance include (i) development of a physical barrier, e.g., melanin protecting from UV-induced damage; (ii) inactivation of metal ions involved in ROS formation; (iii) quenching of excited molecules; (iv) a decrease in pro-oxidant enzyme activities; (v) non-enzymatic neutralization of ROS; and (vi) a specific conversion of ROS to harmless compounds by antioxidant enzymes [36,41]. Antioxidant enzymes, including superoxide dismutase, catalase, and glutathione peroxide, are considered to be the first line of defense against ROS. It is well known that antioxidant enzymes cooperate. The balance between their cellular level and activity is important for cell homeostasis. SOD inactivates the superoxide radical by dismutation into hydrogen peroxide, which in turn is decomposed by CAT and GPx. CAT removes hydrogen peroxide by conversion to hydrogen and oxygen using NADPH as a cofactor, whereas GPx reduces hydrogen peroxide to water with the simultaneous oxidation of glutathione to the disulfide form [28,36,38].

SOD is a family of enzymes residing in extracellular spaces and intracellular compartments. There are two isoforms of SOD in human cells: a cytoplasmatic isoform SOD1 containing copper and zinc and a mitochondrial isoform SOD2 containing manganese [28]. Marklund [42] revealed that the level of SOD in tissues varies depending on the isoform type. The amount of SOD2 was lower and accounted for 20–30% of SOD1. Although SOD1 is constitutively expressed, various repressors and activators of its expression were reported. In contrast to SOD1, the expression of SOD2 is inducible [43].

CAT and GPx cooperate in hydrogen peroxide decomposition, however, CAT is considered to be the major enzyme responsible for the reduction of this reactive molecule in melanocytes [29]. GPx, like SOD, is a family of enzymes. There are eight isoforms of GPx in mammalian cells and the most common is GPx isoform 1 [30]. The induction of CAT and GPx expression is observed in oxidative stress conditions with hydrogen peroxide generation [44,45].

The present study showed that lomefloxacin and moxifloxacin altered the activity and the expression of antioxidant enzymes. Both analyzed FQs decreased SOD activity and expression of SOD1—lomefloxacin at the protein level and moxifloxacin at the mRNA level. Surprisingly, both FQs increased the protein level of SOD2. The enhanced expression of SOD2 may result from the biochemical properties of this isoform. SOD2 is located in the mitochondrial matrix. A major side of superoxide radical generation and its expression is induced in oxidative stress conditions [46].

Lomefloxacin and moxifloxacin greatly differed in impact on hydrogen peroxide-scavenging enzymes. Lomefloxacin alone increased expression and activity of CAT and GPx, whereas moxifloxacin decreased CAT activity and protein and mRNA levels. In the case of GPx we observed that moxifloxacin did not cause significant changes in enzyme activity and protein level, however, it did increase the mRNA level at an established time point. It has been demonstrated that FQs cause alterations in antioxidant enzyme activities and FQ-induced oxidative stress is associated with adverse reactions of these drugs [47]. However, our results indicate dissimilarity in the impact on antioxidant status in pigmented cells between the most and the least phototoxic FQ derivative, which may explain the different phototoxic potential of these antibiotics.

It is well known that UV-A radiation induces oxidative stress in cells, leading to DNA damage. Mouret et al. [48] indicated a higher level of UVA-induced oxidative lesions in melanocytes than keratinocytes. The possible explanation of these observations may be the generation of ROS during melanin synthesis and the lower basal activity of antioxidant defense in melanocytes. The results of the current study indicated alterations of antioxidant status in melanocytes induced by UV-A radiation. The irradiation caused a decrease in catalase activity and in the expression of the enzyme. Moreover, UV-A radiation significantly changed the expression of both tested isoforms of SOD. We observed the up-regulation SOD2 and down-regulation of SOD1. Surprisingly, variations in the protein level of SOD isoforms did not correspond to the change in the enzyme activity. However, the observed UVA-induced modulations of the antioxidant defense system are consistent with the results of other in vitro studies. It was reported that UV-A radiation decreased activities of SOD and catalase, and increased expression of SOD2 [49,50,51,52].

The obtained results showed that UV-A radiation modified the influence of FQs on the antioxidant enzymes in melanocytes. In the case of the impact on SOD, we observed relatively minor differences between lomefloxacin and moxifloxacin. UV-A potentiated the lomefloxacin-induced decrease in SOD activity and in SOD1 expression. In contrast, UV-A did not increase the effect of moxifloxacin on SOD activity. However, despite the lack of differences in enzyme activity between irradiated and non-irradiated cells treated with moxifloxacin, we observed changes in the protein level of SOD1 and SOD2. Non-irradiated cells possessed higher level of SOD1 and SOD2 compared to irradiated melanocytes.

The most important dissimilarity between lomefloxacin and moxifloxacin was the pattern of hydrogen peroxide-scavenging enzyme alterations upon UV-A irradiation. Therefore, we hypothesize that the differences in phototoxic activity between these FQ derivatives may be connected with various hydrogen peroxide production. UV-A radiation enhanced the impact of moxifloxacin on CAT and GPx activity and expression, especially in the protein level. In turn, the exposure of melanocytes to UV-A radiation significantly augmented the lomefloxacin-induced increase in GPx activity and the expression of the enzyme. Moreover, the irradiation reversed the impact of lomefloxacin on the activity and the expression of CAT. The significant changes related to CAT and GPx in melanocytes exposed to lomefloxacin and UV-A radiation might result from the intensification of ROS generation. Overproduction of hydrogen peroxide, which could not be removed by catalase, perhaps due to a decrease in the activity, initially increased the expression and the activity of GPx, and then led to oxidative damage of this enzyme.

## 4. Materials and Methods

### 4.1. Chemicals

Moxifloxacin hydrochloride (Avelox^TM^ solution for IV use containing 400 mg of moxifloxacin per 250 mL in 0.8% saline) was purchased from Bayer Healthcare Pharmaceuticals Inc. (Berlin, Germany). Lomefloxacin hydrochloride, penicillin G, amphotericin B, SIGMAFAST™ Protease Inhibitor Cocktail Tablet, and Phosphatase Inhibitor Cocktail 3, Dulbecco’s phosphate-buffered saline (DPBS) with CaCl_2_ and MgCl_2_, and phosphate buffered saline (PBS) were purchased from Sigma Aldrich Inc. (St. Louis, MO, USA). Neomycin sulfate was obtained from Amara (Kraków, Poland). Trypsin/EDTA solution, growth medium M-254, and a human melanocyte growth supplement-2 (HMGS-2) were purchased from Cascade Biologics/Gibco (Carlsbad, CA, USA). A Superoxide Dismutase Assay Kit, Catalase Assay Kit, and Glutathione Peroxidase Assay Kit were obtained from Cayman Chemical (Ann Arbor, MI, USA). A Pierce BCA Protein Assay Kit and ECL Western Blotting Substrate were obtained from Thermo Fisher Scientific (Waltham, MA, USA).

Immunoblot analysis was performed using GAPDH (14C10) Rabbit mAb, SOD1 (71G8) Mouse mAb, SOD2 (D9V9C) Rabbit mAb, Catalase (D4P7B) Rabbit mAb, and GPx1 (C8C4) Rabbit mAb from Cell Signaling (Danvers, MA, USA), and Anti-Rabbit IgG (A154), Anti-Mouse IgG, Tween-20, RIPA Buffer and PVDF membranes from Sigma-Aldrich Inc. (St. Louis, MO, USA).

RT-qPCR analysis was made using a TRIZOL reagent from Thermo Fisher Scientific (Waltham, MA, USA), OneStep™ PCR Inhibitor Removal Kit from Zymo Research (Irvine, CA, USA), SensiFAST™ SYBR No-ROX kit from Bioline (London, UK), and KiCqStart SYBR Green Primers (SOD1, SOD2, CAT, GPx1, GAPDH) from Sigma-Aldrich Inc. (St. Louis, MO, USA).

### 4.2. Cell Culture

Human epidermal melanocytes, neonatal, darkly pigmented HEMn-DP (Cascade Biologics, Portland, OR, USA) were cultured in an M-254 medium at 37 °C in 5% CO2. The growth medium was supplemented with HMGS-2, neomycin (10 μg/mL), penicillin (100 U/mL), and amphotericin B (0.25 μg/mL). All experiments were performed using cells in passages 5–9.

### 4.3. Cell Treatment and UV-A Exposure

Melanocytes were seeded into a 96-well microplate (2500 cells/well) and into Petri dishes (1,000,000 cells/dish) and preincubated in the growth medium at 37 °C and 5% CO_2_ for 48 h. Then the medium was replaced by lomefloxacin and moxifloxacin solutions and the cells were treated for next 24 h. At the same time, the control samples were cultured in the growth medium. Subsequently, the medium/drug solutions were changed for DPBS and the cells were exposed to UV-A radiation (λ_max_ = 365 nm, 720 μW/cm^2^) for 30 min using a filtered lamp BVL-8.LM (Vilber Lourmat, Collégien, France). The non-irradiated cells had been kept in the dark at 37 °C and 5% CO_2_. Afterward, DPBS was removed and melanocytes were incubated in the culture medium until analysis.

### 4.4. Cell Viability Assay

The cell viability was estimated by WST-1 colorimetric assay. After a 21 h incubation after the irradiation, 10 µl of WST-1 reagent was added to each well and cells were cultured for next 3 h. Subsequently, the absorbance was measured at 440 nm with a reference wavelength of 650 nm using the microplate reader Infinite 200 Pro controlled by the Magellan software. The viability of the melanocytes was expressed as the percentage of the control (untreated cells).

### 4.5. Antioxidant Enzyme Activity Assay

After a 24 h incubation after the irradiation, the melanocytes were trypsinized, suspended in the lysis buffer (lysis buffer consisted of PBS, phosphatase, and protease inhibitors), and lysed by freezing in liquid nitrogen. The activity of the antioxidant enzymes, i.e., superoxide dismutase (SOD), catalase (CAT), and glutathione peroxidase (GPx), was determined in cell lysates using the appropriate spectrophotometric assay kits (Cayman) according to the manufacturer’s instructions. The controls were normalized to 100% for each assay and the enzyme activity in treated melanocytes was expressed as the percentage of the controls.

### 4.6. Western Blot Analysis

Melanocytes were cultured in the growth medium for 24 h after the irradiation. Then the cells were lysed in RIPA buffer containing phosphatase and protease inhibitors. Cell lysates were centrifuged at 12,000 rpm for 10 min at 4 °C for purification from melanin. Afterward, the protein concentrations in the supernatants were determined spectrophotometrically using a Pierce™ BCA Protein Assay Kit. Protein extracts (20 µg/lane) were separated on a 10% SDS-polyacrylamide gel electrophoresis and transferred to PVDF membranes. Subsequently, membranes were incubated for 1 h in blocking buffer (5% non-fat milk Tris-buffered saline with Tween 20). Primary antibodies rabbit anti-GAPDH (1:1000), mouse anti-SOD1 (1:1000), rabbit anti-SOD2 (1:1000), rabbit anti-CAT (1:500), and rabbit anti-GPx1 (1:1000) were diluted in blocking buffer. The analyzed membranes were incubated with the primary antibodies overnight at 4 °C. In the next step, the membranes were kept with appropriate horseradish peroxidase-conjugated secondary antibody (1:10,000 diluted in blocking buffer) for 1.5 h at room temperature. The protein signals were detected using an enhanced chemiluminescence reagent (ECL). Densitometric analysis was conducted using the G:Box Chemi-XT4 Imaging System.

### 4.7. Real-Time Quantitative PCR 

Melanocytes were detached after 8 h of incubation after the irradiation procedure. The total RNAs were extracted using TRIZOL. Then the melanin was removed from RNA extracts by the use of a OneStep™ PCR Inhibitor Removal Kit (Zymo Research, USA) and the total RNA was quantified spectrophotometrically (Denovix DS-11). Quantitative RT-PCR analysis was performed on a real-time fluorescence quantitative PCR instrument (LightCycler^®^ 96 Instrument, South San Francisco, CA, USA) by the use of a SensiFAST™ SYBR No-ROX kit (Bioline) and specific primers, described in Table 1. The PCR parameters were as follows: 45 °C for 10 min, 95 °C for 2 min, followed by 45 cycles of 95 °C for 5 s, 60 °C for 10 s, 72 °C for 1 min, and a final extension for 10 min at 72 °C. The mRNA levels were calculated using the 2^−^^△△^^Ct^ method.

### 4.8. Statistical Analysis

Statistical analysis was performed with GraphPad Prism 6.01 Software. The results are presented as mean values ± SD, calculated from three independent experiments performed in triplicate. The results were analyzed statistically by means of one-way ANOVA (the influence of UV-A radiation or drugs) and two-way ANOVA (the influence of UV-A radiation and drugs) as well as Dunnett’s and Tukey’s multiple comparison tests. Values with *p* < 0.05 were considered statistically significant.

## 5. Conclusions

In our study, we demonstrated for the first time the phototoxic activity of FQs on melanin-containing cells, including drugs’ effects on the antioxidant defense system. Melanin neutralizes ROS and binds xenobiotics, and therefore may protect surrounding cells and tissues from the harmful influence of oxidative damage and drugs. On the other hand, FQ–melanin complex formation may lead to accumulation of these antibiotics and increase their toxicity to melanin-rich tissues.

We determined differences in the reduction of cell viability and alterations of antioxidant enzyme activity and expression in melanocytes exposed to UV-A radiation and lomefloxacin/moxifloxacin, the most and the least phototoxic FQs.

In conclusion, the presented data indicate that the difference in the phototoxic potential between tested FQs may result from the dissimilarity in (i) influence on the antioxidant system, (ii) chemical structure (substitution at C-8 position), and (iii) the ability to form complexes with melanin.

## Figures and Tables

**Table 1:** Nucleotide sequences of primers used in RT-qPCR analysis.

Gene	Forward Primer (5′→3′)	Reverse Primer (5′→3′)
GAPDH	CTTTTGCGTCGCCAG	TTGATGGCAACAATATCCAC
SOD1	GAGCAGAAGGAAAGTAATGG	GATTAAAGTGAGGACCTGC
SOD2	ATCATACCCTAATGATCCCAG	AGGACCTTATAGGGTTTTCAG
CAT	AGAGAAATCCTCAGACACATC	CAGCTTGAAAGTATGTGATCC
GPx1	CTACTTATCGAGAATGTGGC	CAGAATCTCTTCGTTCTTGG

## Abbreviations

CAT	Catalase
DPBS	Dulbecco’s phosphate-buffered saline
ECL	Enhanced Chemiluminescence Reagent
FQs	fluoroquinolones
GPx1	glutathione peroxidase isoform 1
HEMn-DP	human epidermal melanocytes, neonatal, darkly pigmented
HMGS-2	human melanocyte growth supplement-2
PBS	phosphate buffered saline
ROS	reactive oxygen species
SOD	superoxide dismutase
SOD1	superoxide dismutase isoform 1
SOD2	superoxide dismutase isoform 2
